# Supplementary material for: Antibiotic stewardship benchmarking–Using the WHO point prevalence survey of antimicrobial prescribing in a Tertiary Care Public Hospital, Karachi
Source: PLoS One. 2026 Feb 24;21(2):e0342985. doi: 10.1371/journal.pone.0342985 (PMC12931792; doi:10.1371/journal.pone.0342985)
Supplement: S1 Appendix — Form used for collecting patient demographic and clinical data. (DOCX) [file pone.0342985.s001.docx]

Patient Case Report Tool

| Hospital Name: | Research Assistant code: | | | |
| --- | --- | --- | --- | --- |
|  | Patient’s MR No: | | | |
|  | Did patient give consent | - Yes | - No |  |
| Gender: | Patient’s Age (years): | | | |
| - Transfer from other hospital   Yes  No  Unknown   - Previous hospitalization within 90 days   Yes  No  Unknown   - Fever at the time of admission   Yes  No   - History of allergy documented   Yes  No | - Antibiotic administered in Emergency Room (ER)   Yes  No  Not applicable   - Length of Stay till PPS day, Median (Min – Max) - TLC count at admission, Median (Min – Max) x 109/L - Surgery since admission   Yes  No   - Central Venous Catheter   Yes  No   - Peripheral vascular Catheter   Yes  No   - Endotracheal tube   Yes  No   - Urinary Catheter   Yes  No | | | |
| Admission specialty in Hospital (WHO classification) | Hospital admission date: | | | |
| Ward name (DUHS) | Ward admission date: | | | |
| Ward type  Male General Medicine ward Female Surgical ward Adult Intensive Care unit Female General Medicine ward Mixed ward Pediatric Intensive Care Unit Male Surgical ward Cardiology ward | |  | Urology ward |  |
| Patient shifted from another health care facility: ■ Yes ■No | | | | |
| Patient’s hospitalization within 90 days before current admission: ■Yes ■No | | | | |
| Any catheterization on admission ■Yes ■No Insertion date (if yes):  Intubation number:  Intubation type: | | | | |
| Any surgical procedure done after admission:  Type of surgery (If yes): ■ Non- invasive ■Minimally invasive ■invasive | | | |  |
