# Supplementary material for: Antibiotic stewardship benchmarking–Using the WHO point prevalence survey of antimicrobial prescribing in a Tertiary Care Public Hospital, Karachi
Source: PLoS One. 2026 Feb 24;21(2):e0342985. doi: 10.1371/journal.pone.0342985 (PMC12931792; doi:10.1371/journal.pone.0342985)
Supplement: S2 Appendix — Form documenting antibiotic prescriptions, including drug name, dose, route, duration and indication. (DOCX) [file pone.0342985.s002.docx]

Antibiotic Use Form

Patient receives antibiotic at the time of survey: ■No ■Yes (Fill the following form if yes)

No of antibiotics prescribed , No of indications for which antibiotics are prescribed , type of indication (For surgical prophylaxis, include antibiotics administered within 24hrs, before 8a.m. on the survey day)

| **Antibiotic name (generic or brand)** | **Route of administration^1^** | **Indication^2^** | **Type of surgical prophylaxis (If Indication is SP)^3^** | | **If type of SP is SP0, duration of SP0** | **Indication documented** | **Type of treatment (to be filled if indication is CAI or HAI)^4^** | **Diagnosis (site)^5^** | **Start date** | **Dosage per day** | | | | **Is a stop/review order^6^** | **Guidelines compliance^7^** |
| --- | --- | --- | --- | --- | --- | --- | --- | --- | --- | --- | --- | --- | --- | --- | --- |
|  |  |  |  |  |  |  |  |  |  | **No of doses** | **Strength of dose** | **Unit** |  |  |  |
|  | - P - O - I - R | - CAI - HAI - SP - MP - O - UI | - SP0 - SP1 - SP2 - SP3 | | - 1 dose - >1 dose   within 24 hrs   - >1 dose in more than24 hrs. | - Yes - No | - Empirical - Targeted |  |  |  |  |  |  | - Yes - No - UK | ■A  ■B  ■C  ■D  ■E |
|  | - P - O - I - R | - CAI - HAI - SP - MP - O - UI | - SP0 - SP1 - SP2 - SP3 | | - 1 dose - >1 dose   within 24 hrs   - >1 dose in more than24 hrs. | - Yes - No | - Empirical - Targeted |  |  |  |  |  |  | - Yes - No - UK | ■A  ■B  ■C  ■D  ■E |
|  | - P - O - I - R | - CAI - HAI - SP - MP - O - UI | - SP0 - SP1 - SP2 - SP3 | | - 1 dose - >1 dose   within 24 hrs   - >1 dose in more than24 hrs. | - Yes - No | - Empirical - Targeted |  |  |  |  |  |  | - Yes - No - UK | ■A  ■B  ■C  ■D  ■E |
| Sample has been taken for microbiology diagnostic?   - Yes ■No   Sample taken before first dose of antibiotics ■Yes ■No  No of culture tests performed: | | | | Specimen used if sample has been taken (could be >1)   - Respiratory specimen (date/time: ), - urine(date/time: ), ■wound (date/time: ), ■ blood (date/time: ), - other specimen(date/time: )   The results of the culture are communicated to the ward ■Yes ■No | | | | | | | Antimicrobial susceptibility test ordered   - Yes ■No   Are results communicated ■Yes ■No | | | | |
| 1. ***Route of administration: P*** *(Parenteral),* ***O*** *(Oral),* ***I*** *(Inhalation),* ***R*** *(Rectal)* 2. ***Indication: CAI*** *(Community acquired infection)****, HAI (Hospital Acquired Infection), SP*** *(Surgical Prophylaxis),* ***MP (****Medical Prophylaxis)****,O*** *(other)****,UI****(Unknown Indication)* 3. ***Type of surgical prophylaxis: SP0****: If patient receives prophylactic AM before surgery,* ***SP1****:1 dose for surgical prophylaxis,* ***SP2****: Multiple doses on 1 day****, SP3****: Multiple doses on more than 1 day.* 4. ***Type of treatment: Empirical*** *(when antibiotic is used on the basis of physician’s experience before culture test result,* ***Targeted*** *(antibiotic used on the basis on microbiological test results.* 5. *See annex (list of* ***diagnosis site*** *for antibiotic use)* 6. ***Is a stop/review order documented****: Is the stop/review of antibiotic being planned or documented in patient’s notes.(UK=Unknown)* 7. ***Guidelines compliance: A****)No AM guidelines,* ***B****)not compliant,* ***C****) yes compliant,* ***D****) no information,* ***E****)not assessable(>1 indication AM)* | | | | | | | | | | | | | | | |
