# Supplementary material for: Antibiotic stewardship benchmarking–Using the WHO point prevalence survey of antimicrobial prescribing in a Tertiary Care Public Hospital, Karachi
Source: PLoS One. 2026 Feb 24;21(2):e0342985. doi: 10.1371/journal.pone.0342985 (PMC12931792; doi:10.1371/journal.pone.0342985)
Supplement: S3 Appendix — Form for recording microbiological specimen details, culture results, and susceptibility data. (DOCX) [file pone.0342985.s003.docx]

Microbiology Form

| **LABORATORY FINDINGS – MICROBIOLOGY** | | | | | | |
| --- | --- | --- | --- | --- | --- | --- |
| Serial number of Specimen | Specimen type | Date & time of sample taken | Culture Result(Positive/negative/NA | Microorganism | Sensitive Antibiotics | Resistant Antibiotics |
| Specimen 1 |  |  |  |  |  |  |
| Specimen 2 |  |  |  |  |  |  |
| Specimen 3 |  |  |  |  |  |  |
| Specimen 4 |  |  |  |  |  |  |
| Specimen 5 |  |  |  |  |  |  |
