# Supplementary material for: Antibiotic stewardship benchmarking–Using the WHO point prevalence survey of antimicrobial prescribing in a Tertiary Care Public Hospital, Karachi
Source: PLoS One. 2026 Feb 24;21(2):e0342985. doi: 10.1371/journal.pone.0342985 (PMC12931792; doi:10.1371/journal.pone.0342985)
Supplement: S4 Appendix — Checklist used to identify and categorize antimicrobial stewardship opportunities based on predefined criteria including incorrect dosing, spectrum overlap, unnecessary prolonged prophylaxis, absence of indication, inappropriate antibiotic selection and missed IV-to-oral switch. (DOCX) [file pone.0342985.s004.docx]

Common Antibiotic Stewardship Alert

| **Antimicrobial stewardship** | |
| --- | --- |
|  | Not applicable |
|  | Applicable |
|  | Data Missing |
| **Reasons for antimicrobial stewardship** | |
|  | **Antibiotic not indicated** (Antibiotic prescribed without clear clinical evidence of infection or prophylaxis.) |
|  | **Unjustified prolonged duration of therapy** (Antibiotic course extended beyond recommended or clinically necessary duration.) |
|  | **Non-compliance to surgical prophylaxis guidelines** (Surgical prophylaxis does not follow local or international guidelines e.g., timing, agent selection) |
|  | **Extended surgical prophylaxis** (Prophylaxis continued beyond 24 hours post-surgery without justification.) |
|  | **Antibiotic on discharge not needed** (Antibiotic prescribed at discharge without clear indication or continuation criteria.) |
|  | **Microorganism resistant to antibiotic used** (Antibiotic used despite microbiology results showing resistance) |
|  | **Inappropriate choice of antibiotic** (Antibiotic selected is not suitable for the suspected or confirmed pathogen, spectrum too broad, or not aligned with guidelines.) |
|  | **Restricted antibiotics use** (Use of antibiotics that require special authorization from ID or are reserved for specific situations and cannot be used without proper approval.) |
|  | **Narrow spectrum options available** (A broader-spectrum antibiotic was used when a narrower, appropriate alternative was available.) |
|  | **Incorrect dose** (Dose is inappropriate for patient’s age, weight, renal function, or infection severity.) |
|  | **Contraindication to use of current antibiotic** (Antibiotic used despite known patient allergy, intolerance, or drug interaction.) |
|  | **Overlapping spectrum** (Two or more antibiotics with similar spectra prescribed unnecessarily, leading to duplication.) |
|  | **Other** (Any additional stewardship concern not covered by the above categories.) |
