# Supplementary material for: Antibiotic stewardship benchmarking–Using the WHO point prevalence survey of antimicrobial prescribing in a Tertiary Care Public Hospital, Karachi
Source: PLoS One. 2026 Feb 24;21(2):e0342985. doi: 10.1371/journal.pone.0342985 (PMC12931792; doi:10.1371/journal.pone.0342985)
Supplement: S5 Tables — Table A – showing demographic and clinical details. Table B – detailing descriptive data of the wards. Table C – providing description of route of administration, frequency and site of antibiotics administration. (DOCX) [file pone.0342985.s005.docx]

Supplementary Tables

Table A. Demographic and clinical details of patients at admission 2

| **Variables** | | **n=186** | **%** | **95% CI** |
| --- | --- | --- | --- | --- |
| Patient belong in which group of age | Neonates(<=28 days) | 5 | 2.7 | 0.9-6.2 |
|  | Infant (01 month -12 month) | 2 | 1.1 | 0.1-3.9 |
|  | Nursery (1-3 year) | 4 | 2.2 | 0.6-5.5 |
|  | Kindergarten (3-7 years) | 5 | 2.7 | 0.9-6.2 |
|  | Elementary school(7-13 years) | 6 | 3.2 | 1.2-6.9 |
|  | Adolescence (13-17 years) | 6 | 3.2 | 1.2-6.9 |
|  | Youth (17-21 years) | 6 | 3.2 | 1.2-6.9 |
|  | Crown of youth (21-25years). | 8 | 4.3 | 1.9-8.3 |
|  | Young age (25 to 44) | 47 | 25.3 | 19.3-32.0 |
|  | Middle age (44-60) | 46 | 24.7 | 18.8-31.5 |
|  | Elderly age (60-75) | 43 | 23.1 | 17.2-29.8 |
|  | Senile age (75-90) | 8 | 4.3 | 1.9-8.3 |
| Ward type ( as per WHO) | Adult high risk ward | 44 | 23.7 | 17.8-30.4 |
|  | Adult intensive care unit | 35 | 18.8 | 13.5-25.5 |
|  | Adult medical ward | 32 | 17.2 | 12.1-23.4 |
|  | Adult surgical ward | 9 | 4.8 | 2.2-9.0 |
|  | Mix ward | 46 | 24.7 | 18.8-31.5 |
|  | Neonatal Intensive care unit | 5 | 2.7 | 0.9-6.2 |
|  | Pediatric high risk ward | 2 | 1.1 | 0.1-3.9 |
|  | Pediatric intensive care unit | 3 | 1.6 | 0.3-4.6 |
|  | Pediatric medical ward | 10 | 5.4 | 2.6-9.7 |
| Number of diagnosis on single patient | Single diagnosis | 131 | 70.4 | 63.3-76.8 |
|  | Twice diagnosis | 37 | 19.9 | 14.4-26.4 |
|  | Triple diagnosis | 15 | 8.1 | 4.6-12.9 |
|  | Multiple diagnoses( >3) | 3 | 1.6 | 0.3-4.6 |
| Admission specialty in hospital (as per WHO classification) | GOGYN | 11 | 5.9 | 3.0-10.3 |
|  | ICUMED | 25 | 13.4 | 8.9-19.2 |
|  | ICUNEO | 5 | 2.7 | 0.9-6.2 |
|  | ICUPED | 3 | 1.6 | 0.3-4.6 |
|  | MEDCARD | 9 | 4.8 | 2.2-9.0 |
|  | MEDENDO | 1 | 0.5 | 0.0-2.9 |
|  | MEDGAST | 19 | 10.2 | 6.3-15.5 |
|  | MEDGEN | 27 | 14.5 | 9.8-20.4 |
|  | MEDHEMA | 15 | 8.1 | 4.6-12.9 |
|  | MEDHEMBMT | 3 | 1.6 | 0.3-4.6 |
|  | MEDHEP | 7 | 3.8 | 1.7-7.6 |
|  | MEDNEPH | 15 | 8.1 | 4.6-12.9 |
|  | MEDNEU | 11 | 5.9 | 3.0-10.3 |
|  | MEDONCO | 3 | 1.6 | 0.3-4.6 |
|  | MEDPNEU | 3 | 1.6 | 0.3-4.6 |
|  | PEDGEN | 10 | 5.4 | 2.6-9.7 |
|  | SURGEN | 4 | 2.2 | 0.6-5.5 |
|  | SURNEU | 1 | 0.5 | 0.0-2.9 |
|  | SURORTO | 4 | 2.2 | 0.6-5.5 |
|  | SUROTH | 1 | 0.5 | 0.0-2.9 |
|  | SURTRANS | 4 | 2.2 | 0.6-5.5 |
|  | SURURO | 5 | 2.7 | 0.9-6.2 |
| Patient transfer from another hospital | Yes | 6 | 3.2 | 1.2-6.9 |
|  | No – direct admission | 176 | 94.6 | 90.3-97.4 |
|  | Unknown | 4 | 2.2 | 0.6-5.5 |
| Patient hospitalized within 90 days before current admission | Yes | 7 | 3.8 | 1.5-7.6 |
|  | No | 172 | 92.5 | 87.8-95.8 |
|  | Unknown | 7 | 3.8 | 1.5-7.6 |
| Does Patient Febrile at time of admission? (See history at time of admission for symptoms of fever reported) | Yes | 62 | 33.3 | 26.6-40.6 |
|  | No | 124 | 66.7 | 59.4-73.4 |
| Does History of allergy recorded in Patient Profile? | Yes | 48 | 25.8 | 19.6-32.8 |
|  | No | 138 | 74.2 | 67.2-80.4 |
| Use of antimicrobial by patient before admission(see medication reconciliation or past medication history) | Yes | 5 | 2.7 | 0.9-6.2 |
|  | No | 56 | 30.1 | 23.6-37.2 |
|  | Unknown | 125 | 67.2 | 60.0-73.8 |
| Antibiotic administered in ER | Yes | 72 | 38.7 | 31.7-46.1 |
|  | No | 46 | 24.7 | 18.8-31.5 |
|  | Not applicable(not admitted through ER) | 68 | 36.6 | 29.7-43.9 |
| Length of stay at time of PPS survey | Median (Min – Max) | 3 | 1 – 45 |  |
| TLC count at the time of admission | Median (Min – Max) | 9.8 | 1 – 96 |  |
| Last creatinine at the time of PPS | Median (Min – Max) | 0.92 | 1 – 9 |  |
| Surgery since admission | Yes | 30 | 16.1 | 11.2-22.2 |
|  | No | 156 | 83.9 | 77.8-88.8 |
| Any central vascular catheter | Yes | 8 | 4.3 | 1.9-8.3 |
|  | No | 178 | 95.7 | 91.7-98.1 |
| Any peripheral vascular catheter | Yes | 163 | 87.6 | 82.0-92.0 |
|  | No | 23 | 12.4 | 8.0-18.0 |
| Any endotracheal tube | Yes | 5 | 2.7 | 0.9-6.2 |
|  | No | 181 | 97.3 | 93.8-99.1 |
| Any urinary catheter | Yes | 94 | 50.5 | 43.2-57.9 |
|  | No | 92 | 49.5 | 42.1-56.8 |
| Patient received antimicrobials at the time of survey? | Yes | 155 | 83.3 | 77.2-88.4 |
|  | No | 31 | 16.7 | 11.6-22.8 |
| All percentages calculated from total patients (n=186). ER = Emergency Room; TLC = Total Leukocyte Count. Specialty codes: First 3 letters indicate department (MED=Medical, SUR=Surgical, PED=Pediatric, ICU=Intensive Care Unit), remaining letters indicate subspecialty. Complete specialty names are: GOGYN (Gynecology & Obstetrics), ICUMED (Medical ICU), ICUNEO (Neonatal ICU), ICUPED (Pediatric ICU), MEDCARD (Medical Cardiology), MEDENDO (Medical Endocrinology), MEDGAST (Medical Gastroenterology), MEDGEN (Medical General), MEDHEMA (Medical Hematology), MEDHEMBMT (Medical Hematology-Bone Marrow Transplant), MEDHEP (Medical Hepatology), MEDNEPH (Medical Nephrology), MEDNEU (Medical Neurology), MEDONCO (Medical Oncology), MEDPNEU (Medical Pulmonology), PEDGEN (Pediatric General), SURGEN (Surgical General), SURNEU (Surgical Neurology), SURORTO (Surgical Orthopedics), SUROTH (Surgical Other), SURTRANS (Surgical Transplant), SURURO (Surgical Urology). | | | | |

Table B. Descriptive data of ward Point Prevalence Survey (PPS)

| **Total** | **N** | **Mean** | **SD** | **Minimum** | **Maximum** |
| --- | --- | --- | --- | --- | --- |
| Total Patients In Wards(present at 8.am on the day of survey) | 224 | 8.4 | 3.7 | 2.0 | 15.0 |
| Total Number Of Eligible Patients In The Ward At 8.AM on the day of survey) | 186 | 6.6 | 3.86 | 1.0 | 16.0 |
| Total Number Of Non Eligible Patients In The Ward At 8.Am On The Day Of Survey) | 38 | 1.75 | 2.25 | 0.0 | 8.0 |
| Table-10 reports the PPS ward assessment descriptive, on average 8.4 (SD=±3.7) patients present at 8 A.M on the day of survey, 6.6 (SD=±3.8) number of eligible patients in the ward at 8 A.M on the day of survey, 1.75 (SD=±2.2) number of non-eligible patients in the ward at 8 A.M on the day of survey.  SD = Standard Deviation. Data collected according to WHO Point Prevalence Survey methodology v1.1. | | | | | |

Table C. Description on route of administration, frequency and site of antibiotics administration

|  | N | % | 95% CI |
| --- | --- | --- | --- |
| Route of administration (N=222) |  |  |  |
| Oral | 24 | 10.8 | 7.1-15.7 |
| Parenteral | 198 | 89.2 | 84.3-92.9 |
| If Parenteral, Type |  |  |  |
| Intravenous intermittent | 177 | 89.4 | 84.2-93.3 |
| Intravenous continuous infusion | 20 | 10.1 | 6.3-15.1 |
| Other | 1 | 0.5 | 0.0-2.8 |
| If Parenteral, oral switch done, (N=204) |  |  |  |
| Yes | 6 | 2.9 | 1.1-6.3 |
| No | 195 | 95.6 | 91.7-97.9 |
| Unknown | 3 | 1.5 | 0.3-4.3 |
| Indication or Diagnosis (SITE) for Prescribed Antimicrobial (N=77) |  |  |  |
| BJ-SSI | 1 | 1.3 | 0.0-7.0 |
| BJ-O | 1 | 1.3 | 0.0-7.0 |
| BRON | 1 | 1.3 | 0.0-7.0 |
| CNS | 14 | 18.2 | 10.4-28.6 |
| CSEP | 7 | 9.1 | 3.7-17.8 |
| CVS | 6 | 7.8 | 2.9-16.2 |
| CYS | 2 | 2.6 | 0.3-9.1 |
| GI | 31 | 40.3 | 29.2-52.0 |
| OBGY | 14 | 18.2 | 10.4-28.6 |
| Status of antibiotic on day of Survey (N=218) |  |  |  |
| Continued to Date | 216 | 99.1 | 96.8-99.9 |
| Stop | 2 | 0.9 | 0.1-3.2 |
| Duration of Antibiotics (Days) [Mean ±SD] | 3.35 | ±2.8 |  |
| Frequency of Antibiotics (N=218) |  |  |  |
| Q12H | 72 | 33.0 | 26.9-39.6 |
| Q24H | 47 | 21.6 | 16.3-27.6 |
| Q6H | 7 | 3.2 | 1.3-6.5 |
| Q8H | 81 | 37.2 | 30.8-43.9 |
| STAT | 7 | 3.2 | 1.3-6.5 |
| Q48H | 4 | 1.8 | 0.4-4.6 |
| Dosage per day (Median, Min – Max) | 2 | 1 – 4 |  |
| Is a Stop/Review order documented? |  |  |  |
| Yes | 219 | 99.1 | 96.8-99.9 |
| No | 2 | 0.9 | 0.1-3.2 |
| Denominators vary for different parameters as indicated. Q12H = every 12 hours; Q24H = every 24 hours; Q6H = every 6 hours; Q8H = every 8 hours; STAT = single dose administration. BJ-SSI = Bone and joint surgical site infection; CNS = Central nervous system; CSEP = Catheter-related sepsis; CVS = Cardiovascular system; CYS = Cystitis; GI = Gastrointestinal; OBGY = Obstetrics and gynecology. | | | |
