# Supplementary material for: Antibiotic stewardship benchmarking–Using the WHO point prevalence survey of antimicrobial prescribing in a Tertiary Care Public Hospital, Karachi
Source: PLoS One. 2026 Feb 24;21(2):e0342985. doi: 10.1371/journal.pone.0342985 (PMC12931792; doi:10.1371/journal.pone.0342985)
Supplement: Attachment — Submitted filename: Response to Reviewers (point by point).docx [file pone.0342985.s007.docx]

**Detailed point-by-point response to reviewers**

**Manuscript ID:** PONE-D-25-59189
**Title:** Antibiotic Stewardship Benchmarking - Using the WHO Point Prevalence Survey of Antimicrobial Prescribing in a Tertiary Care Public Hospital, Karachi
**To:** Dr. Mabel Kamweli Aworh (Academic Editor, *PLOS ONE*) and the esteemed Reviewers

**Date:** 10 January 2026

Dear Dr. Aworh and Reviewers,

We are profoundly grateful for the opportunity to revise and resubmit our manuscript. We sincerely thank you for the exceptional time and expertise invested in evaluating our work. The collective feedback from the editor and all the reviewers was incisive, constructive, and instrumental in identifying critical areas for improvement. We have approached this major revision with the seriousness it deserves, undertaking a comprehensive restructuring and refinement of the manuscript.

This letter provides a detailed, point-by-point response to every comment and requirement. We have made extensive revisions, all of which are transparently highlighted in the uploaded **‘Revised Manuscript with Track Changes’** document. A clean, formatted version is provided as the **‘Manuscript’** file.

We believe the revised manuscript is now substantially strengthened in its methodological transparency, analytical clarity, narrative focus, and alignment with both the WHO-PPS framework and *PLOS ONE*’s publication standards. We are hopeful it now merits your positive consideration.

Sincerely,

**Syed Shaukat Ali Muttaqi Shah, PhD**
On behalf of all co-authors:
Tehreem Ansari, Beenish Syed, Mehwish Rizvi, Fakhsheena Anjum, Tehrim Fatima, Farah Saeed

**Part 1: Response to the Editor’s Specific Major Issues:**

The editor’s summary of major issues provided an excellent roadmap for our revision. We addressed each with substantive changes.

**Editor’s Major Issue 1: Undefined Key Terms**

- **Comment:** “The Introduction repeatedly mentions antimicrobial stewardship (AMS) and Point Prevalence Survey (PPS) without defining them.”
- **Our Response:** We agree that these are core concepts requiring clear definition for a broad readership. We have inserted concise, functional definitions directly into the introductory narrative. These interventions improve rationale antibiotics use by, measuring the use of antibiotics, promoting proper selection of appropriate antibiotics regimen, including suitable dose, duration and preferred route of administration, without compromising patient outcome.
- **Action Taken & Location:**
  1. **Antimicrobial Stewardship Program (ASP):** Added on page 4, lines 75-79: “ASPs is defined as a systemic and coordinated set of interventions aim to optimize the use of antimicrobial agents…”
  2. **Point Prevalence Survey (PPS):** Added on page 4, lines 94-97: “A PPS is a standardized method used in healthcare settings to assess microbial use and prescribing patterns at a specific point of time. Its primary goal is to observe prescribing patterns and compare them with the established guidelines of WHO or CDC. PPS is frequently used to establish benchmarks for practice and evaluate effectiveness of AMS programs”
- **Rationale:** These definitions, placed immediately after the terms are introduced, provide essential context without disrupting the narrative flow, enhancing accessibility for readers from diverse backgrounds.

**Editor’s Major Issue 2: Methodological Clarity**

- **Comment:** Concerns regarding patient inclusion logic, missing statistical procedures, undefined acronyms, and undefined stewardship criteria.
- **Our Response:** We have rewritten the part of methodology and expanded the Methods section (pages 5-8) to eliminate ambiguity and provide full transparency.
- **Action Taken & Location:**
  1. **Patient Inclusion Clarity:** On page 6, lines 125-127, we now explicitly state: “In accordance with the WHO PPS protocol, patients who were temporarily absent from the ward (e.g., for medical imaging, endoscopy, or surgery) were included only if they were admitted to the ward and expected to return later on the survey day.” This directly aligns with WHO-PPS v1.1 criteria.
  2. **Statistical Procedures:** A new subsection on data management and analysis was added (page 7, lines 163-175). It details:
     - **Data Entry & Validation:** “All data extracted from patient records were entered into a structured spreadsheet. A data validation check was performed by a second researcher on a 10% random sample to ensure accuracy and completeness.”
     - **Handling Missing Data:** “Missing data points e.g., undocumented antibiotic stop date, were reconciled by re-reviewing the primary medical record, and were recorded as ‘unknown’ and excluded from analysis to maintain data integrity.”
     - **Descriptive Analysis:** “Descriptive statistics were used to summarize the data: frequencies and percentages are reported for categorical variables... Continuous variables... are presented as mean ± Standard Deviation (SD) if normally distributed, or as median with interquartile range (IQR)...” We also clarify, “No comparative inferential statistics were employed, as the study design was purely a descriptive point prevalence survey.”
  3. **Definition of All Acronyms:** Every acronym (e.g., HDU, ICU, MEDGAST, MEDNEPH, TLC, AWaRe) is now spelled out in full at first use in both the main text and table/figure footnotes.
  4. **Stewardship Criteria:** A dedicated paragraph on page 7, lines 149-158 now describes the criteria: “Stewardship opportunities were identified and categorized using criteria adapted from standard ASP principles. Categories included: over prescribing... incorrect dose... overlapping spectrum...” The complete, operationalized checklist is provided in S4 Appendix.

**Editor’s Major Issue 3: Results Section Issues**

- **Comment:** Results contain WHO interpretations; classification rules should be in Methods; lacks a standard WHO-PPS style summary.
- **Our Response:** This was a critical structural flaw. We have purified the Results section from the interpretations and added the requested PPS summary table.
- **Action Taken & Location:**
  1. **Removed Interpretation:** All evaluative language (e.g., “reflects a potential area for strengthening stewardship”) has been stripped from the Results. The text now presents findings neutrally (e.g., “The detailed distribution of antimicrobial agents... is shown in Table 3”).
  2. **Moved AWaRe Description to Methods:** The explanation of the AWaRe classification (“Access=first-line, Watch=monitored, Reserve=last-resort”) has been moved from the Results to the Methods section, page 7, lines 141-146.
  3. **Added WHO-PPS Summary Table:** The most significant addition is Table 5: WHO-PPS summary of key antibiotic use indicators (page 18-19). This table concisely presents the core metrics expected from a PPS: overall prevalence (83.3%), leading indications (CAI 55.5%), AWaRe distribution (Watch 80.8%), surgical prophylaxis >24h rate (5.0%), parenteral administration rate (89.2%), and IV-to-oral switch rate (2.9%). This provides an immediate, standardized snapshot of the findings.

**Editor’s Major Issue 4: Discussion Inconsistencies**

- **Comment:** Inaccurate claim of “equal gender,” contradictory claims about being the first PPS in Pakistan, mismatched percentages, problematic subtitles, and misplaced limitations.
- **Our Response:** We have performed a line-by-line review and rewrite of the Discussion to correct errors and improve flow.
- **Action Taken & Location:**
  1. **Clarified Novelty and Context:** We have refined the narrative to accurately position our study. The Introduction now states our aim is to fill a gap in data from “major public tertiary care hospitals in Karachi specifically” (page 5, lines 102-103). “This study therefore contributes to the limited but growing body of WHO-PPS data from Pakistan by providing a detailed snapshot of antibiotic use in a public tertiary care hospital in Karachi, a setting underrepresented in the national AMS literature” (page 5, lines 108-110). The Discussion opening acknowledges we “add a contemporary data point to the growing evidence from Pakistan, which includes prior surveys...” (page 22, line 335-337). This resolves the contradiction.
  2. **Corrected Gender Statement**: On page 22, line 341, the text now accurately reads: “The gender distribution and mean age as shown in “Table 1”, are comparable to cohorts of other PPS conducted in Pakistan, suggesting our findings are generalizable within similar settings.
  3. **Verified All Percentages:** All numerical values in the Discussion have been cross-referenced with tables and figures to ensure perfect consistency.
  4. **Removed Subtitles, Created Narrative Flow:** All subheadings (e.g., “4.1 Patient characteristics”) have been deleted. Rewritten the Discussion in a continuous logical format that interprets findings thematically, moving from patient profile to prevalence, antibiotic choices, routes, diagnostics, and finally to implications and limitations.
  5. **Relocated Limitations:** The study limitations have been consolidated into a dedicated, final paragraph of the Discussion (page 29, lines 490-502), as per journal convention.

**Editor’s Major Issue 5: Reference Currency**

- **Comment:** “Please ensure that 80% of the references are within the last 5 years.”
- **Our Response:** We have undertaken a major update of the reference list.
- **Action Taken:** We have replaced older, non-essential references with recent, high-impact literature from 2020-2025, particularly on PPS in LMICs, AWaRe classification, and ASP implementation. Of the 65 final references, 53 (81.5%) are from 2020-2025. We retained a few seminal older papers (e.g., the 2019 WHO PPS manual, key historical policy statements) where their foundational importance warranted it.

**Part 2: Detailed response to reviewer comments**

We are deeply thankful to each reviewer for their insightful critiques, which have pushed us to elevate the quality of our work significantly.

**Reviewer #1**

**Comment 1: “The analysis is descriptive... I recommend including confidence intervals, comparative analysis between ward types... no statistical correlation between antibiotic choice and culture results.”**

- **Our Response:** We thank the reviewer for this suggestion to enhance the analytical depth. We agree such analyses could be informative. However, the WHO Point Prevalence Survey methodology is explicitly designed as a descriptive, benchmarking tool. Its primary objective is to provide a “snapshot” of prescribing patterns at a single point in time to identify areas for quality improvement, not to test hypotheses or establish correlations. Implementing comparative statistics (e.g., between wards or against culture results) would require a different study design with a priori sample size calculations powered for such comparisons.
- **Action Taken:** To address the spirit of this comment, we have:
  1. Confidence intervals (CIs) were calculated using the Wilson score method for binomial proportions, we have now added **95% CIs for all key proportions** throughout the manuscript (e.g., Tables 1, 2, 5) and result sections to indicate precision. Also we have edited the method section accordingly mentioning the calculation of CIs (page 8, lines 171-173).
  2. **Clarified the Study Design** in the Methods: We explicitly state, “No comparative inferential statistics were employed, as the study design was purely a descriptive point prevalence survey” (page 8, lines 173-174).
  3. **Enhanced Discussion of Drivers:** While we cannot provide statistical correlations, we have expanded the Discussion (page 25) to logically explore the *relationship* between high empirical therapy (99.2%), low culture yield (64.8%), and the resultant overuse of Watch-group antibiotics. We frame this as a “critical failure in diagnostic stewardship” that *drives* irrational prescribing, providing the conceptual link the reviewer sought.

**Comment 2: “On the interpretation of the AWaRe findings... the paper does not go further to clarify why Access drugs are not being chosen. Without understanding the likely drivers, stewardship recommendations will remain generic.”**

- **Our Response:** This is an exceptionally valuable point. We have substantially expanded this section to move from simply reporting the high Watch-group use to analyzing its systemic drivers.
- **Action Taken & Location:** We have added a new analytical passage in the Discussion on pages 24, lines 376-397. We now hypothesize several key drivers:
  - **Formulary and Perceived Reliability:** “...carbapenems and advanced-spectrum β-lactams dominate the hospital formularies because of their higher perceived reliability and ease of use.”
  - “Several factors drive the over-reliance on Watch group. First, although Access agents were available in the hospital formulary, they were underutilized compared to broad-spectrum antibiotics, indicating gap in prescriber awareness of AWaRe, lack of restriction policies and perceived therapeutic reliability encourage their empirical use.”
  - **Diagnostic Deficiencies:** “The extensive use of empiric therapy (99.2%) along with high negative culture rates (64.8%) reflects a grey area, where lack of timely microbiology results encourages use of broad-spectrum antibiotics.”
  - **Awareness and Structural Gaps:** “Although Access agents were available in the hospital, they were underutilized... indicating a gap in prescriber awareness of AWaRe... Absence of local guidelines and formulary restrictions provide no safeguard against this pattern.”
- **Impact:** This transforms the discussion from a generic statement (“Watch use is high”) to a root-cause analysis, making our subsequent stewardship recommendations in Table 9 (e.g., prospective audit, diagnostic stewardship, access restrictions) directly targeted and more compelling.

**Comment 3: “In the Microbiology section, the finding that 64.8% of cultures showed no growth is striking, but you could further explore possible causes, as this would help clarify how diagnostic processes might influence prescribing behavior.”**

- **Our Response:** We agree this is a pivotal finding that warrants exploration. A high ‘no growth’ rate is often a proxy for problems in diagnostic stewardship.
- **Action Taken & Location:** We have integrated this analysis into the Discussion section on page 22, lines 426-432. We link it directly to the empirical prescribing pattern:
  - “Prolonged microbiology turnaround time, specimens collected after start of antibiotic therapy, and limited access to proper microbiological facilities for identifying the pathogen and their susceptibility encourage clinicians to default to broad-spectrum antibiotics rather than waiting for the culture guided use of antibiotics...”
  - “This high rate of empiric therapy is not just a prescribing pattern but the root cause resulting in the overuse of Watch-group antibiotics...”
- **Impact:** By framing the low culture yield as a cause rather than just a finding, we strengthen the argument for one of our key recommended interventions: enhancing diagnostic stewardship (Table 9), which includes improving pre-analytical phases like specimen collection timing and investing in rapid diagnostics.

**Reviewer #2**

We thank Reviewer #2 for their positive assessment of the manuscript’s core structure and for providing precise, actionable feedback for improvement.

**Comment 1 (Recommendations 1, 2, 3, 7): Editorial and Presentation Issues**

- **Our Response:** We have meticulously addressed each of these points to enhance professionalism and clarity.
- **Action Taken & Location:**
  - **Table Footnotes:** Every table (1, 2, 3, 4, 5, 6, 7, 8, 9) now includes a “Note:” section defining abbreviations (e.g., CAI, HAI, AWaRe, SD) and explaining any specific details (e.g., “Percentages may exceed 100% due to multiple specimens per patient” in Table 6).
  - **Typographical Corrections:** Corrected “Urinary Cather” to “Catheter” in Table 1. Corrected “adult medial” to “adult medical” on page 12. Numerous other spelling and grammatical errors throughout the text have been corrected.
  - **Scientific Nomenclature:** In Table 7, “E. coli” has been updated to “*Escherichia coli*” and “Klebsiella prunemoniae” to “*Klebsiella pneumoniae*” to ensure uniformity and correctness.
  - **Supplementary Information Clarity:** S4 Appendix now clearly lists and defines each stewardship criterion (e.g., “Over Prescribing: Antibiotic use without a clear clinical indication documented in the notes”).

**Comment 2 (Recommendation 4): “Expand the discussion to provide deeper analysis of the findings and remove repetitive reporting of results.”**

- **Our Response:** This was a central focus of our revision. The previous Discussion largely restated the Results. We have completely rewritten it.
- **Action Taken:** The new Discussion (pages 22-29) avoids simple repetition of results, although brief references to key findings (e.g., 83.3% prevalence, high Watch use) remain in the discussion to provide context, but the section now:
  - **Contextualizes** findings within the Pakistani and LMIC landscape.
  - **Analyzes Root Causes** (e.g., defensive prescribing culture, diagnostic gaps, and absence of guidelines) as detailed in responses to other reviewers.
  - **Explores Implications** for AMR and patient safety.
  - **Synthesizes** findings to build a coherent argument for urgent, multi-faceted stewardship interventions.
    The narrative now interprets rather than reports.

**Comment 3 (Recommendations 5 & 6): “Update the reference list to incorporate more literature published within the last five years. Correct the formatting of references.”**

- **Our Response:** We have conducted a thorough reference update and formatting check.
- **Action Taken:** As reported in response to the Editor, 81.5% of references are now from 2020-2025. We have added recent, relevant studies from Pakistan, India, Bangladesh, Africa, and global PPS literature. All references have been verified and formatted according to PLOS ONE’s Vancouver style guidelines.

**Reviewer #3**

We are grateful to Reviewer #3 for their comprehensive and rigorous critique, which closely mirrored the editor’s concerns and confirmed the areas needing major revision. Their feedback was invaluable.

**Comment 1 (Major Issue - Definitions):** Aligned with Editor’s Issue 1. Addressed as above (see Editor’s Major Issue 1).

**Comment 2 (Major Issue - Methodological Clarity):** Aligned with Editor’s Issue 2. Addressed in full (see Editor’s Major Issue 2), including defining SAP, ONCO, MED, ESBL, AWaRe and detailing stewardship criteria.

**Comment 3 (Major Issue - Results):** Aligned with Editor’s Issue 3. Addressed by purifying the Results, moving AWaRe description to Methods, and adding Table 5: WHO-PPS summary (see Editor’s Major Issue 3).

**Comment 4 (Major Issue - Discussion Inconsistencies & Novelty):**

- **Our Response:** We have corrected the specific inconsistencies and, crucially, strengthened the statement of the study’s rationale and novelty.
- **Action Taken & Location:**
  1. **Gender & Comparisons Corrected:** As detailed in response to the Editor (Issue 4).
  2. **Enhanced Rationale:** The Introduction's final paragraph (page 5, lines 102-110) has been rewritten to clearly articulate the novelty: "However data from major public tertiary care hospitals in Karachi specifically remains sparse... This study therefore aim to apply WHO-PPS methodology in a major public sector hospital in Karachi... the study seeks to fill a significant gap and generate locally relevant data to inform the development of tailored stewardship interventions (as subsequently outlined in Table 9), institutional guidelines, and diagnostic improvements."

**Comment 5 (Minor Issues):** All addressed.

- **Abstract Sample Clarity:** The abstract now specifies “186 inpatients (adults and children across medical, surgical and critical care wards)” (page 2, line 40-41).
- **Figure Labeling:** All figures are numbered sequentially (Fig 1, Fig 2...), have descriptive captions, and legends are decluttered.
- **Duplicate Ethics:** The ethics statement now appears only once, in the Methods.
- **Typos/Terminology/Acronyms:** Corrected and defined throughout the manuscript.

**Reviewer #4**

We thank Reviewer #4 for their overall positive assessment and for identifying a key methodological refinement.

**Comment: “The minor addition that is necessary for the methods section is including the time of microbiological sample collection to Appendix 3. This determines how culture and sensitivity results are interpreted. Were the samples collected before antibiotics or during antibiotics use?”**

- **Our Response:** This is an excellent and practically important suggestion. The timing of specimen collection relative to antibiotic administration is a critical factor in interpreting culture yield and susceptibility results.
- **Action Taken:** We have modified the S3 Appendix (Microbiology data form) to include a dedicated field for recording the “Date and Time of Specimen Collection” and, in S2 Appendix (Antibiotics use Form), a checkbox or field to indicate the “Relationship to Antibiotic Therapy: Sample take Before first dose of antibiotics [ ] Yes [ ]No and specific timing of the sample collection.”
- **Rationale:** While the current PPS is a snapshot and this data wasn’t captured retrospectively, as the exact timing of microbiology sample collection was not systematically documented in patient medical records, revising the data collection tool is a direct, impactful outcome of this study. It will significantly enhance the quality of future PPS audits and diagnostic stewardship analyses at our institution, allowing for a proper assessment of how empirical therapy undermines microbiological diagnosis. We have acknowledged this limitation in the Discussion section, noting that the absence of precise timing information may have influenced culture.

**CONCLUSION**

The revision process has been thorough and transformative. We have:

1. **Corrected all factual errors and inconsistencies.** Gender claim, novelty clarity, percentage inconsistencies and headings in the discussion sections etc
2. **Greatly enhanced methodological transparency and rigor.**
3. **Restructured the manuscript** to adhere to standard reporting formats (e.g., adding the WHO-PPS summary table).
4. **Elevated the Discussion** from descriptive to analytical, exploring root causes and implications.
5. **Updated the literature** to reflect the current state of knowledge.
6. **Fully complied** with all of *PLOS ONE*’s formatting and policy requirements.

We believe the revised manuscript presents a clearer, stronger, and more valuable contribution to the literature on antimicrobial stewardship in LMIC settings. We are deeply appreciative of the guidance provided and hope that our revisions have satisfactorily addressed all points raised.

Thank you once again for your time and consideration and look forward for the positive outcome of the review.

Respectfully submitted,
